# Genetic Study of Severe Prolonged Lymphopenia in Multiple Sclerosis Patients Treated With Dimethyl Fumarate

**DOI:** 10.3389/fgene.2019.01039

**Published:** 2019-11-04

**Authors:** Dipen Sangurdekar, Chao Sun, Helen McLaughlin, Katherine Ayling-Rouse, Normand E. Allaire, Michelle A. Penny, Paola G. Bronson

**Affiliations:** ^1^Translational Genome Sciences, Translational Biology, Biogen, Cambridge, MA, United States; ^2^Human Target Validation Core, Translational Biology, Biogen, Cambridge, MA, United States; ^3^Envision Pharma Group, Horsham, United Kingdom

**Keywords:** multiple sclerosis, lymphopenia, dimethyl fumarate, pharmacogenomics, transcriptomics

## Abstract

In delayed-release dimethyl fumarate (DMF)-treated patients, absolute lymphocyte count (ALC) often declines in the first year and stabilizes thereafter; early declines have been associated with development of severe prolonged lymphopenia (SPL). Prolonged moderate or severe lymphopenia is a known risk factor for progressive multifocal leukoencephalopathy (PML); DMF-associated PML is very rare. It is unknown whether genetic predictors of SPL secondary to DMF treatment exist. We aimed to identify genetic predictors of reduced white blood cell (WBC) counts in DMF-treated multiple sclerosis (MS) patients. Genotyping (*N* = 1,258) and blood transcriptional profiling (*N* = 1,133) were performed on MS patients from DEFINE/CONFIRM. ALCs were categorized as: SPL, < 500 cells/µL for ≥6 months; moderate prolonged lymphopenia (MPL), < 800 cells/µL for ≥6 months, excluding SPL; mildly reduced lymphocytes, < 910 cells/µL at any point, excluding SPL and MPL; no lymphopenia, ≥910 cells/µL. Genome-wide association, HLA, and cross-sectional gene expression studies were performed. No common variants, HLA alleles, or expression profiles clinically useful for predicting SPL or MPL were identified. There was no overlap between genetic peaks and genetic loci known to be associated with WBC. Gene expression profiles were not associated with lymphopenia status. A classification model including gene expression features was not more predictive of lymphopenia status than standard covariates. There were no genetic predictors of SPL (or MPL) secondary to DMF treatment. Our results support ALC monitoring during DMF treatment as the most effective way to identify patients at risk of SPL.

## Introduction

Multiple sclerosis (MS) is a demyelinating neurodegenerative disease. Delayed-release dimethyl fumarate (DMF [Tecfidera^®^]) is an approved oral disease modifying therapy for patients with relapsing–remitting MS (RRMS) and has a favorable benefit–risk profile (Fox et al., 2012; Gold et al., 2012). Lymphocyte decline is a known effect of DMF treatment; on average, patients experience a 30% decrease in absolute lymphocyte count (ALC) during the first year of DMF treatment (Fox et al., 2016). In clinical trials, ∼5% of patients treated with DMF experienced severe lymphopenia (ALC <0.5 × 10^9^/L) at least once during treatment (Fox et al., 2012; Gold et al., 2012). Very rare cases of progressive multifocal leukoencephalopathy (PML) in the setting of lymphopenia have occurred in patients treated with DMF, predominantly in the context of moderate prolonged lymphopenia (MPL; ≥0.5 to <0.8 × 10^9^/L) or severe prolonged lymphopenia (SPL; <0.5 × 10^9^/L), persisting for ≥6 months (Sweetser et al., 2013; Dammeier et al., 2015). Prescribing guidelines vary according to local labels, but most recommend intermittent monitoring of absolute lymphocyte count (ALC) and treatment interruption in patients with SPL persisting for more than 6 months. The objective of this study was to perform a genome-wide association study (GWAS) and transcriptomic profiling to identify clinically relevant genomic risk factors for developing SPL or MPL secondary to DMF.

## Methods

### Genetic Analysis

Whole blood samples were collected from a subset of DMF-treated patients with RRMS from the DEFINE/CONFIRM trials under the main study informed consent, at baseline, and at other time points during the study (Fox et al., 2012; Gold et al., 2012). Genomic DNA was prepared from whole blood by Covance Central Labs using standard laboratory protocols. Whole-genome single-nucleotide polymorphism (SNP) genotyping was performed in the Biogen genetics research lab in compliance with Biogen research guidelines but not under Good Laboratory Practice Regulations. DNA concentrations were normalized to 50 ng/μL and 200 ng DNA of each sample was used for genotyping (Human Omnichip 2.5-V1.2; Illumina, Inc., San Diego, CA).

### Genome-Wide Association Study (GWAS) Statistical Analysis

Patients with RRMS were divided into four phenotypic groups for analysis based on the following ALC criteria: SPL, ALC <0.5 × 10^9^/L (corresponding to National Cancer Institute Common Toxicity Criteria [CTCAE] grade 3) for ≥6 months; MPL, ALC 0.5 – <0.8 × 10^9^/L (corresponding to CTCAE grade 2) for ≥6 months; mildly reduced lymphocytes, (grade 1) ALC 0.8 – <0.91 × 10^9^/L (lower limit of normal [LLN]); and no lymphopenia, ALC always >LLN.

A GWAS was performed on ∼1 million DNA variants using logistic regression analysis of SPL versus no lymphopenia, and SPL or MPL versus no lymphopenia (i.e., excluding the mildly reduced lymphocytes phenotype). The analyses were run two ways: by adjusting for two significant ancestry principal components; and adjusting for the top two principal components and baseline ALC. We adjusted for baseline ALC because in this dataset it was associated with severe prolonged lymphopenia [odds ratio (OR) 5.2, *p* = 5 × 10^−6^] and with SPL or MPL (OR 4.2, *p* = 1 × 10^−16^). The randomized controlled trial design of the study had a treatment washout period prior to the start of the trial and therefore covariates such as previous treatments were not adjusted for. Analyses were conducted in PLINK v1.9.

We conducted χ^2^ tests of association on genotypes to estimate a genomic inflation factor (λ_GC_) based on the median χ^2^ for genotyped variants, adjusted for ancestry. *P* values were plotted in a quantile-quantile plot and in a Manhattan plot. The genome-wide significance threshold (*p* < 5e−8) was based on the standard Bonferroni correction for a GWAS. Power was estimated using Power for Genetic Analysis (v2) to plot the detectable OR versus variant allele frequency. The GWAS had 80% power to detect an OR of 4.0 in the SPL versus no lymphopenia cohort, and 80% power to detect an OR of 1.8 in the SPL or MPL versus no lymphopenia cohort (**Figure S1**).

### Classical HLA Allele Statistical Analysis

We imputed seven classical human leukocyte antigen (HLA) alleles (HLA-DRB1, HLA-DQB1, HLA-DQA1, HLA-DPB1, HLA-A, HLA-B, HLA-C) with HIBAG 1.8.3 (Zheng et al., 2014). Analyses were adjusted for baseline ALC (false discovery rate <0.05).

### Gene Expression Analysis

Whole blood in PAXgene tubes was collected with informed consent from all patients in DEFINE/CONFIRM at baseline and at various time points during the study. RNA samples were prepared using the PreAnalytiX PAXgene 96 blood kit according to the manufacturer’s protocol at Expression Analysis Inc. (Morrisville, NC). Following RNA extraction and QC, genome-wide gene expression data was generated using the Affymetrix GeneChip Human Genome U133 Plus 2.0 Array (Thermo Fisher Scientific, Waltham, MA). The array contained 54,675 probes targeting most annotated genes in the human genome. Raw expression data was subject to rigorous QC and normalization. All array files (CEL) were normalized using guanine cytosine robust multiarray analysis to remove array processing-related signals. To remove observed effects related to upstream sample processing, expression data was normalized for RNA processing batch, initial yield of RNA from samples, final yield of labeled RNA, RNA integration number (an index for RNA quality), and RNA degradation 5′-3′ slope. While adjusting for these effects, we also controlled for treatment arm and time point.

To identify whether baseline cross-sectional profiles might reveal a prognostic signature for lymphopenia, all patients from the DEFINE/CONFIRM trials with subsequent lymphopenia status were considered, including patients in the placebo arms of the trials who switched to treatment in the ENDORSE extension study (a cross-sectional study of lymphocyte changes in long-term patients with RRMS treated with DMF) and developed lymphopenia (Gold et al., 2017). Because there were fewer samples with no lymphopenia that had gene expression profiles available, we included patients with “mildly reduced lymphocytes” in our transcriptomic analysis. For patients crossing over from the placebo arm, the week 96 sample expression data from DEFINE/CONFIRM was considered for the cross-sectional analysis. Gene expression profile cross-sectional analyses were performed and adjusted for ancestry, ALC, age, sex, and disease duration using the *limma* package (Ritchie et al., 2015) available from *R* statistical software.

A statistical model for predicting SPL or MPL status from no lymphopenia or mildly reduced lymphocytes status was built. Two models, one with age, sex, ALC, and disease duration (base model) and another including gene expression features to the covariates (full model) were built. For both models, logistic regression was used to predict class labels and performance metrics were estimated using 10-fold cross-validation. For the full model, feature selection within each fold of cross-validation was performed using an internally 5-fold cross-validated elastic-net algorithm implemented in the *glmnet* package (Friedman et al., 2010) available from *R* statistical software.

Standard GWAS QC methods were applied in this study. For variant QC, we applied an Illumina Gen Train score threshold of ≥0.7, a genotyping rate of ≥0.98, a minor allele frequency of >0.01, a genotyping rate of ≥0.99, and a Hardy–Weinberg equilibrium threshold of 1 × 10^−4^. We also excluded variants with a differential call rate. For sample QC, we dropped samples with heterozygosity ≥6 standard deviations, samples with cryptic relatedness, and samples that were ancestry outliers (based on default SMARTPCA algorithm).

## Results

### GWAS

**Figure 1:**
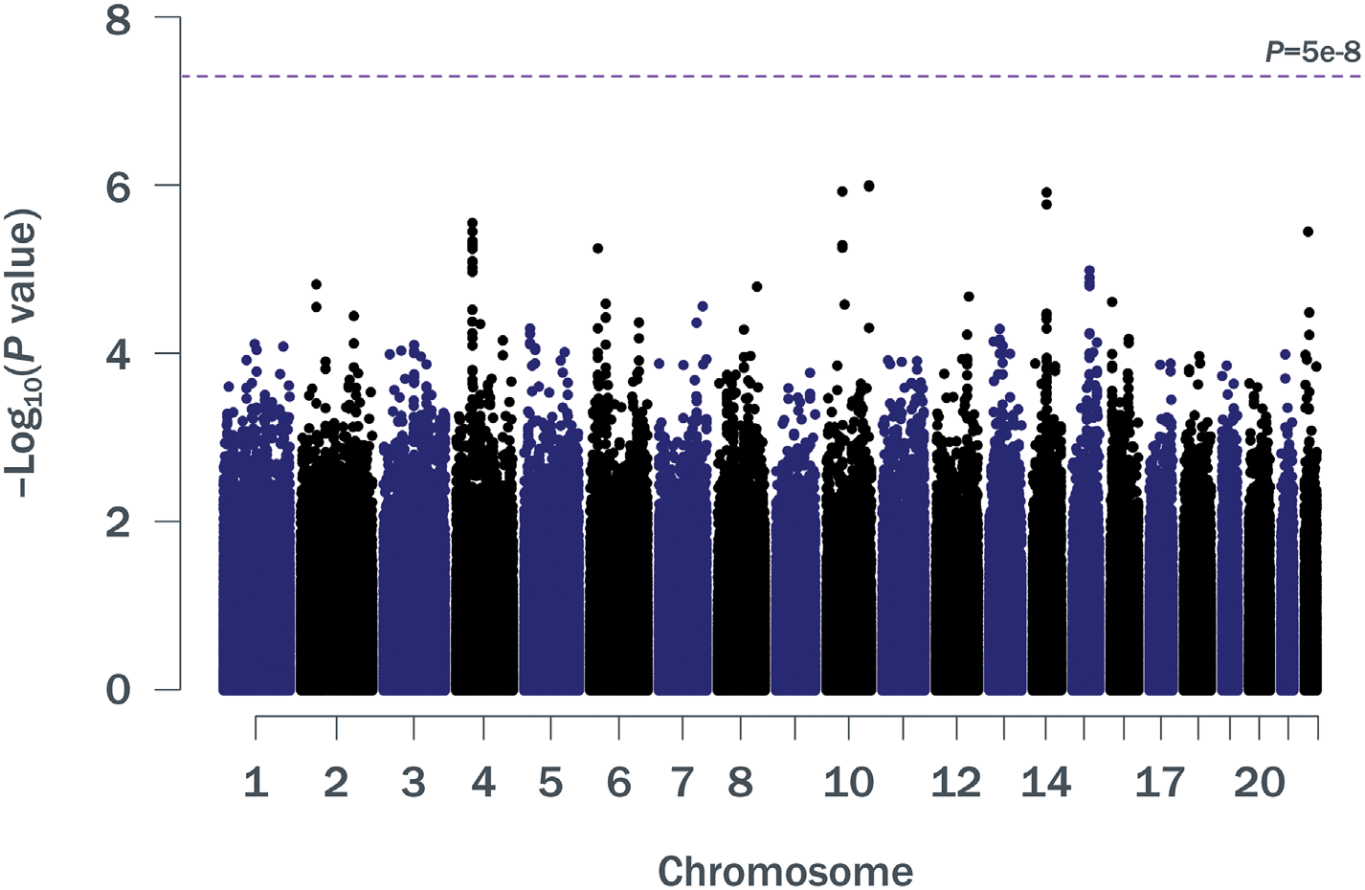
Manhattan plot of *p* values for ∼1 million SNPs (minor allele frequency ≥0.05) in a GWAS of SPL or MPL (*n* = 206) versus no lymphopenia (*n* = 1,052). GWAS, genome-wide association study; MPL, moderate prolonged lymphopenia; SNP, single nucleotide polymorphism; SPL, severe prolonged lymphopenia.

Patient baseline characteristics by ALC criteria are shown in **Table 1**. After QC, there were 1,016,018 unique variants for GWAS analysis, from a total of 42 samples available from the SPL cohort, 164 from the MPL cohort, and 1,052 samples from the no lymphopenia controls. None of the DNA variants tested reached genome-wide significance (*p* < 5e−8) in the analysis of SPL versus no lymphopenia or in the analysis of SPL or MPL (**Table 2**, **Figure 1**, **Table S1**, **Figures S2**–**S4**). Results did not differ when baseline ALC was excluded as a covariate (data not shown). Further, results did not differ when adjusting for age and sex. Development of severe, prolonged lymphopenia and moderate, prolonged lymphopenia were not associated with age or sex.

**Table 1:** Samples included in the GWAS.

Lymphopenia status	SPL	MPL	No lymphopenia	Total
Definition	ALC <0.5 × 10^9^/L for ≥6 months	ALC <0.8 × 10^9^/L for ≥6 months^a^	ALC always >0.91 × 10^9^/L (LLN)	
Number analyzed	42	164	1,052	1,258
Age, mean (SD)	43.7 (8.3)	40.9 (7.8)	37.8 (9.1)	38.4 (9.0)
Female, n (%)	35 (83)	121 (74)	738 (70)	894 (71)
Years since diagnosis, mean (SD)	5.2 (5.23)	5.5 (5.74)	5.1 (5.21)	5.2 (5.28)
Relapses in prior year, mean (SD)	1.4 (0.70)	1.3 (0.56)	1.3 (0.64)	1.3 (0.64)
EDSS, mean (SD)	2.75 (1.32)	2.45 (1.15)	2.42 (1.22)	2.44 (1.22)
Mean baseline ALC (SD)	1.64 (0.43)	1.68 (0.71)	2.07 (0.60)	

ALC, absolute lymphocyte count; EDSS, Expanded Disability Status Scale; GWAS, genome-wide association study; LLN, lower limit of normal; MPL, moderate prolonged lymphopenia; SPL, severe prolonged lymphopenia. All samples were of European ancestry.

a Excludes patients in the SPL category (ALC <0.5 × 10^9^/L for ≥6 months).

**Table 2:** Number of samples and genetic variants (SNPs) included in the genetic study (GWAS) of SPL and MPL. The genomic control inflation factor is close to 1, which indicates that ancestry was properly controlled for.

	Lymphopenia, *n*	No lymphopenia, *n*	Total, *n*	Genomic control inflation factor, λ_GC_	SNPs genotyped, *n*
SPL vs. no lymphopenia	42	1,052	1,094	1.00	1,016,025
SPL or MPL vs. no lymphopenia	206	1,052	1,258	0.99	1,016,018

GWAS, genome-wide association study; MPL, moderate prolonged lymphopenia; SNP, single nucleotide polymorphism; SPL, severe prolonged lymphopenia.

GWAS results for variants in the major histocompatibility complex region on chromosome 6 did not identify any strong HLA associations (**Figure S5**): the greatest association for the SPL versus no lymphopenia analysis was at the intergenic variant rs2647088 (*p* = 2.4 × 10^−3^; OR 2.1; 95% confidence interval [CI] 1.3–3.5; chr6:32,681,518). This SNP is located between HLA-DQB1 and HLA-DQA2. The greatest association for the SPL or MPL versus no lymphopenia analysis was at rs382259 (*p* = 2.5 × 10^−5^; OR 1.7; 95% CI 1.3–2.2; chr6:32,209,027; **Figure S6**). The peak on chromosome 6 from the SPL GWAS was different from the peak on chromosome 6 for the SPL + MPL GWAS (**Table S2**).

We also examined the overlap between our peak GWAS association regions with published GWAS on white blood cell counts or lymphocyte counts. Based on the GWAS catalog, there were 8 studies on white blood cell counts, reporting 24 regions in total (38 peak SNPs) (Soranzo et al., 2009; Kamatani et al., 2010; Nalls et al., 2011; Reiner et al., 2011; Crosslin et al., 2012; Kong and Lee, 2013; Li et al., 2013; Keller et al., 2014). Of these peak SNPs, 17 were present in our study and none had *p* < 0.10 in the same direction. The proportion of total variance explained by associated variants was estimated to be 16–24% (Reiner et al., 2011). There was 1 GWAS specifically on lymphocyte counts, and it reported four nonsignificant peak regions, none of which overlapped with the peak regions identified in this study (Okada et al., 2011).

### Gene Expression Analysis

**Figure 2:**
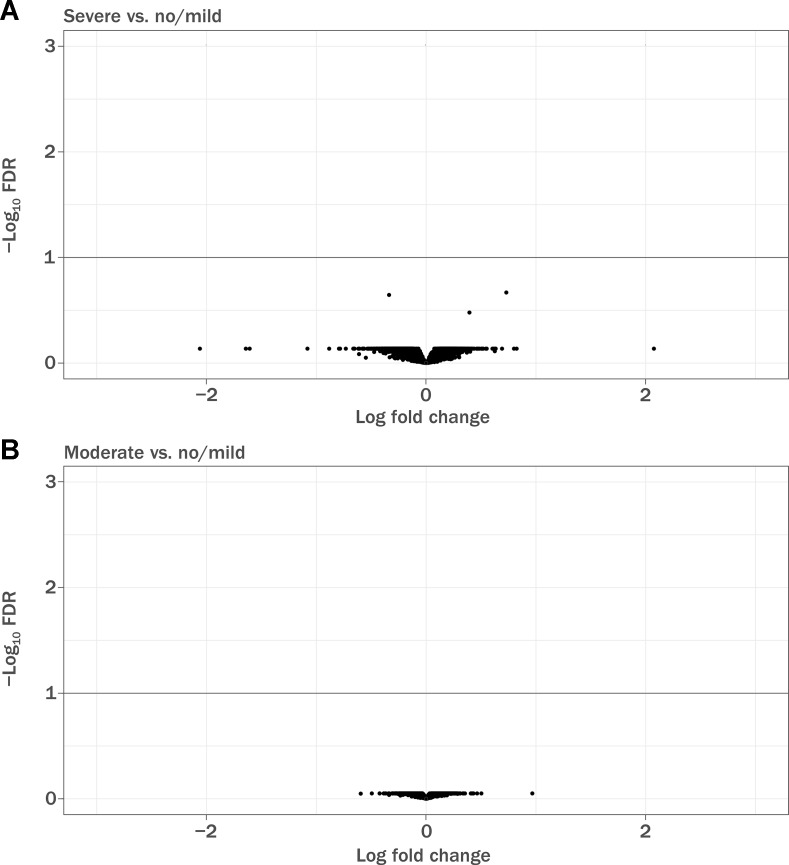
Volcano plot demonstrating differentially expressed genes in baseline RNA samples. FDR, false discovery rate. **(A)** Patients with severe prolonged lymphopenia (*n* = 42) versus no lymphopenia or mildly reduced lymphocytes (*n* = 942) and **(B)** patients with moderate prolonged lymphopenia (*n* = 149) versus no lymphopenia or mildly reduced lymphocytes (*n* = 942). Sample numbers correspond to **Table 4**.

Of 1,460 patients with documented lymphopenia status, 1,133 also had gene expression data (**Table 3**). Demographic characteristics of the samples used have been previously described (Fox et al., 2012; Gold et al., 2012). Note that the GWAS sample numbers in **Table 1** differ from the gene expression sample numbers in **Table 3**. When adjusted for covariates, baseline gene expression profiles were not associated with lymphopenia status (false discovery rate <10%; **Figure 2**). The array used in the gene expression analysis contained probes specific for the glutathione-S-transferase T1 (GSTT1) locus, and it was observed that baseline expression level of GSTT1 was not associated with lymphopenia status of baseline ALC (**Figure S7**).

**Table 3:** Samples included in the gene expression analysis.

Lymphopenia status	DMF twice daily	DMF 3 times daily	Placebo to extension	Total
SPL	15	9	18	42
MPL	54	40	55	149
No lymphopenia or mildly reduced lymphocytes^a^	284	315	343	942

DMF, delayed-release dimethyl fumarate; MPL, moderate prolonged lymphopenia; SPL, severe prolonged lymphopenia.

a Absolute lymphocyte count (ALC) <0.91 × 10^9^/L (lower limit of normal) at any point, but ALC not <800 cells/μL for ≥6 months.

An exploratory classification model (full model) including gene expression features was not more predictive (mean accuracy 68%) of lymphopenia status than the base model including the clinical covariates age, sex, ALC, and disease duration alone (mean accuracy 68%; **Figure S8**).

## Discussion

This genome-wide analysis of DNA variants and RNA expression profiles did not provide additional insight into risk factors relevant to the development of SPL in patients treated with DMF. Given the power of this genetic study we could conclude that there are no common variants of large effect that would be clinically useful in predicting SPL. From patient baseline characteristics, older age and lower baseline ALC appeared to be associated with lymphopenia (**Table 1**) but groups were not powered to detect a difference. However, age was not fully predictive; younger patients also developed lymphopenia while some older patients did not. Therefore, our results support the use of ALC monitoring as an effective strategy to identify patients who may be at risk for developing prolonged lymphopenia, for patients of all ages. Gene expression profiling analysis identified no differentially expressed genes in patients with RRMS with SPL and MPL compared with patients with mildly reduced lymphocytes or no lymphopenia when age, sex, ALC, and disease duration were used as covariates. Since gene expression in whole blood is heavily influenced by ALC levels, this suggests that transcript level changes are a surrogate for ALC changes on treatment. The addition of gene expression features to an exploratory classification model to predict the eventual lymphopenia status of patients did not significantly improve the prediction performance of a model above that which comprised covariates.

A small study that genotyped a deletion in GSTT1 in patients with psoriasis conducted by Gambichler et al. (2014) suggested that a patient homozygous for the *0/*0 deletion at the locus had a marked reduction in lymphocyte count on treatment with oral fumaric acid esters (Gambichler et al., 2014). The presence of the deletion significantly correlated with enzyme activity. Such small genetic studies are likely to identify false positive results. In the current study, no variants were genotyped in the GSTT1 gene region on chromosome 22 (24,376,135–24,384,284); therefore, it was not possible to directly evaluate the genetic association with this gene. However, the gene expression data did contain probes for the GSTT1 locus and no difference in gene expression was observed across the different categories of lymphopenia, suggesting that the null allele may not be impacting lymphocyte count.

The major histocompatibility complex on chromosome 6, a gene-dense region of immunological importance that includes the HLA loci, has been associated with autoimmune disease as well as adverse drug events for a variety of therapies (Pirmohamed et al., 2015). For these reasons, we took a closer look at our results in this region, even though they did not reach genome-wide significance. The peak in our SPL + MPL GWAS was at rs382259 in the MHC class III region, near *NOTCH4*. This overlaps with a GWAS signal for Crohn’s disease (P = 1.4 × 10^−9^, OR = 2.3), as well as a secondary signal for ulcerative colitis chr6: 32,280,470–32,282,252 (IMAGEN et al., 2009). It also overlaps with a primary signal for systemic lupus erythematosus that was reported in this region (IMAGEN et al., 2009), however conditional haplotype analyses indicate that the signal is tagging the SLE risk allele HLA-DRB1*03:01 (Morris et al., 2012). The major histocompatibility complex peaks on chromosome 6 differed between the two studies. The imputed classical HLA alleles were not significantly associated after correcting for multiple testing.

In conclusion, no common genetic variants or imputed HLA alleles were associated (at genome-wide significance) with SPL or SPL or MPL in this exploratory analysis of DNA samples collected in the DEFINE/CONFIRM pivotal phase 3 clinical trials. Overall, we saw no overlap between nonsignificant peak GWAS regions observed in this study (**Table 4**), and the GWAS associated with white blood cell counts in published studies (Soranzo et al., 2009; Kamatani et al., 2010; Nalls et al., 2011; Reiner et al., 2011; Crosslin et al., 2012; Kong and Lee, 2013; Li et al., 2013; Keller et al., 2014). A cross-sectional analysis of baseline gene expression profiles did not identify any differentially expressed genes that would be clinically useful in predicting SPL. This exploratory genome-wide molecular profiling analysis has not provided additional insight into the risk factors relevant to the development of SPL or MPL in patients treated with DMF. These data support current ALC monitoring labeling guidelines as the most effective method for identifying patients of all ages who may be at risk of developing prolonged lymphopenia.

**Table 4:** Peak association regions in the SPL or MPL GWAS.

SNP	Allele	*p* Value	OR (95% CI)	Chromosome	Region	Gene
rs12249496	A	9.9e−7	2.2 (1.6–3.0)	10	130,670,196–130,686,938	
rs17417060	G	1.2e−6	2.1 (1.6–2.9)	10	45,412,777–45,446,791	*TMEM72*
kgp3442760	A	1.2e−6	1.9 (1.5–2.5)	14	62,635,965–62,652,220	*TMEM72-AS1*
kgp5383559	A	2.8e−6	2.0 (1.5–2.8)	4	53,586,179–53,739,262	*ERVMER34-1 LOC152578*
kgp11318955	A	4.5e−6	1.8 (1.4–2.3)			*RASL11B* *SCFD2*
kgp4348860	A	3.5e−6	2.4 (1.7–3.6)	22	28,149,693–28,150,815	*MN1*
rs870204	A	5.5e−6	2.0 (1.5–2.7)	10	45,458,485	*RASSF4*
kgp2538268	G	5.6e−6	1.8 (1.4–2.3)	6	19,078,368–19,128,940	*LOC101928519*

GWAS, genome-wide association study; MPL, moderate prolonged lymphopenia; OR, odds ratio; SNP, single nucleotide polymorphism; SPL, severe prolonged lymphopenia.

## Data Availability Statement

DEFINE and CONFIRM were registered with ClinicalTrials.gov (NCT00420212, NCT00451451). Requests for data supporting this manuscript should be submitted to the Biogen Clinical Data Request Portal (www.biogenclinicaldatarequest.com). Publicly available datasets were analyzed in this study. This data can be found here: GSE55851, GSE29312, GSE29332, GSE17718, GSE6034, GSE38537, GSE33615, GSE57259, GSE19080.

## Author Contributions

DS, MP, PB participated in the interpretation of the study results and in the drafting and critical revision of the manuscript. CS, HM, and NA participated in the drafting and critical revision of the manuscript. KA-R participated in the drafting and revision of the manuscript. All authors approved the final version of the manuscript.

## Conflict of Interest

DS, NA, HM, CS, MP, and PB are employees of and hold stock/stock options in Biogen. KA-R is an employee of and holds stock/stock options in Envision Pharma Group. The study was funded/supported by Biogen. Biogen was involved in the study design, data collection, data analysis, and preparation of the manuscript.

## References

[B1] Crosslin D. R. McDavid A. Weston N. Nelson S. C. Zheng X. Hart E. (2012). Genetic variants associated with the white blood cell count in 13,923 subjects in the eMERGE Network. Hum. Genet. 131 (4), 639–652. 10.1007/s00439-011-1103-9 22037903 PMC3640990

[B2] Dammeier N. Schubert V. Hauser T. K. Bornemann A. Bischof F. (2015). Case report of a patient with progressive multifocal leukoencephalopathy under treatment with dimethyl fumarate. BMC Neurol. 15, 108. 10.1186/s12883-015-0363-8 26152311 PMC4495627

[B3] Fox R. J. Chan A. Gold R. Phillips J. T. Selmaj K. Chang I. (2016). Characterizing absolute lymphocyte count profiles in dimethyl fumarate-treated patients with MS: patient management considerations. Neurol. Clin. Pract. 6 (3), 220–229. 10.1212/CPJ.0000000000000238 27347439 PMC4909524

[B4] Fox R. J. Miller D. H. Phillips J. T. Hutchinson M. Havrdova E. Kita M. (2012). Placebo-controlled phase 3 study of oral BG-12 or glatiramer in multiple sclerosis. N. Engl. J. Med. 367 (12), 1087–1097. 10.1056/NEJMoa1206328 22992072

[B5] Friedman J. Hastie T. Tibshirani R. (2010). Regularization paths for generalized linear models via coordinate descent. J. Stat. Software 33 (1), 1–22. 10.18637/jss.v033.i01 PMC2929880 20808728

[B6] Gambichler T. Kreuter A. Susok L. Skrygan M. Rotterdam S. Hoxtermann S. (2014). Glutathione-S-transferase T1 genotyping and phenotyping in psoriasis patients receiving treatment with oral fumaric acid esters. J. Eur. Acad. Dermatol. Venereol. 28 (5), 574–580. 10.1111/jdv.12137 23489263

[B7] Gold R. Arnold D. L. Bar-Or A. Hutchinson M. Kappos L. Havrdova E. (2017). Long-term effects of delayed-release dimethyl fumarate in multiple sclerosis: interim analysis of ENDORSE, a randomized extension study. Mult. Scler. 23 (2), 253–265. 10.1177/1352458516649037 27207449 PMC5418934

[B8] Gold R. Kappos L. Arnold D. L. Bar-Or A. Giovannoni G. Selmaj K. (2012). Placebo-controlled phase 3 study of oral BG-12 for relapsing multiple sclerosis. N. Engl. J. Med. 367 (12), 1098–1107. 10.1056/NEJMoa1114287 22992073

[B9] International MHC and Autoimmunity Genetics Network (IMAGEN) Rioux J. D. Goyette P. Vyse T. J. Hammarstrom L. Fernando M. M. A. (2009). Mapping of multiple susceptibility variants within the MHC region for 7 immune-mediated diseases. Proc. Natl. Acad. Sci. U. S. A. 106 (44), 18680–18685. 10.1073/pnas.0909307106 19846760 PMC2773992

[B10] Kamatani Y. Matsuda K. Okada Y. Kubo M. Hosono N. Daigo Y. (2010). Genome-wide association study of hematological and biochemical traits in a Japanese population. Nat. Genet. 42 (3), 210–215. 10.1038/ng.531 20139978

[B11] Keller M. F. Reiner A. P. Okada Y. van Rooij F. J. Johnson A. D. Chen M. H.. (2014). Trans-ethnic meta-analysis of white blood cell phenotypes. Hum. Mol. Genet. 23 (25), 6944–6960. 10.1093/hmg/ddu401 25096241 PMC4245044

[B12] Kong M. Lee C. (2013). Genetic associations with C-reactive protein level and white blood cell count in the KARE study. Int. J. Immunogenet. 40 (2), 120–125. 10.1111/j.1744-313X.2012.01141.x 22788528

[B13] Li J. Glessner J. T. Zhang H. Hou C. Wei Z. Bradfield J. P. (2013). GWAS of blood cell traits identifies novel associated loci and epistatic interactions in Caucasian and African-American children. Hum. Mol. Genet. 22 (7), 1457–1464. 10.1093/hmg/dds534 23263863 PMC3657475

[B14] Morris D. L. Taylor K. E. Fernando M. M. Nititham J. Alarcon-Riquelme M. E. Barcellos L. F. (2012). Unraveling multiple MHC gene associations with systemic lupus erythematosus: model choice indicates a role for HLA alleles and non-HLA genes in Europeans. Am. J. Hum. Genet. 91 (5), 778–793. 10.1016/j.ajhg.2012.08.026 23084292 PMC3487133

[B15] Nalls M. A. Couper D. J. Tanaka T. van Rooij F. J. Chen M. H. Smith A. V. (2011). Multiple loci are associated with white blood cell phenotypes. PLoS Genet. 7 (6), e1002113. 10.1371/journal.pgen.1002113 21738480 PMC3128114

[B16] Okada Y. Hirota T. Kamatani Y. Takahashi A. Ohmiya H. Kumasaka N. (2011). Identification of nine novel loci associated with white blood cell subtypes in a Japanese population. PLoS Genet. 7 (6), e1002067. 10.1371/journal.pgen.1002067 21738478 PMC3128095

[B17] Pirmohamed M. Ostrov D. A. Park B. K. (2015). New genetic findings lead the way to a better understanding of fundamental mechanisms of drug hypersensitivity. J. Allergy Clin. Immunol. 136 (2), 236–244. 10.1016/j.jaci.2015.06.022 26254050 PMC4534769

[B18] Reiner A. P. Lettre G. Nalls M. A. Ganesh S. K. Mathias R. Austin M. A. (2011). Genome-wide association study of white blood cell count in 16,388 African Americans: the continental origins and genetic epidemiology network (COGENT). PLoS Genet. 7 (6), e1002108. 10.1371/journal.pgen.1002108 21738479 PMC3128101

[B19] Ritchie M. E. Phipson B. Wu D. Hu Y. Law C. W. Shi W. (2015). *limma* powers differential expression analyses for RNA-sequencing and microarray studies. Nucleic Acids Res. 43 (7), e47. 10.1093/nar/gkv007 25605792 PMC4402510

[B20] Soranzo N. Spector T. D. Mangino M. Kuhnel B. Rendon A. Teumer A.(2009). A genome-wide meta-analysis identifies 22 loci associated with eight hematological parameters in the HaemGen consortium. Nat. Genet. 41 (11), 1182–1190. 10.1038/ng.467 19820697 PMC3108459

[B21] Sweetser M. T. Dawson K. T. Bozic C. (2013). Manufacturer's response to case reports of PML. N. Engl. J. Med. 368 (17), 1659–1661. 10.1056/NEJMc1300283 23614605

[B22] Zheng X. Shen J. Cox C. Wakefield J. C. Ehm M. G. Nelson M. R. (2014). HIBAG—HLA genotype imputation with attribute bagging. Pharmacogenomics J. 14 (2), 192–200. 10.1038/tpj.2013.18 23712092 PMC3772955

