# Supplementary material for: Genetic Study of Severe Prolonged Lymphopenia in Multiple Sclerosis Patients Treated With Dimethyl Fumarate
Source: Front Genet. 2019 Nov 4;10:1039. doi: 10.3389/fgene.2019.01039 (PMC6844186; doi:10.3389/fgene.2019.01039)
Supplement: Supplementary file 1 [file Presentation_1.pdf]

Supplementary Material

1 Supplementary Figures and Tables

1.1 Supplementary Figures

Supplementary Figure S1 GWAS power calculations to identify clinically relevant effects in **(A)** SPL versus no lymphopenia and **(B)** SPL or MPL versus no lymphopenia.

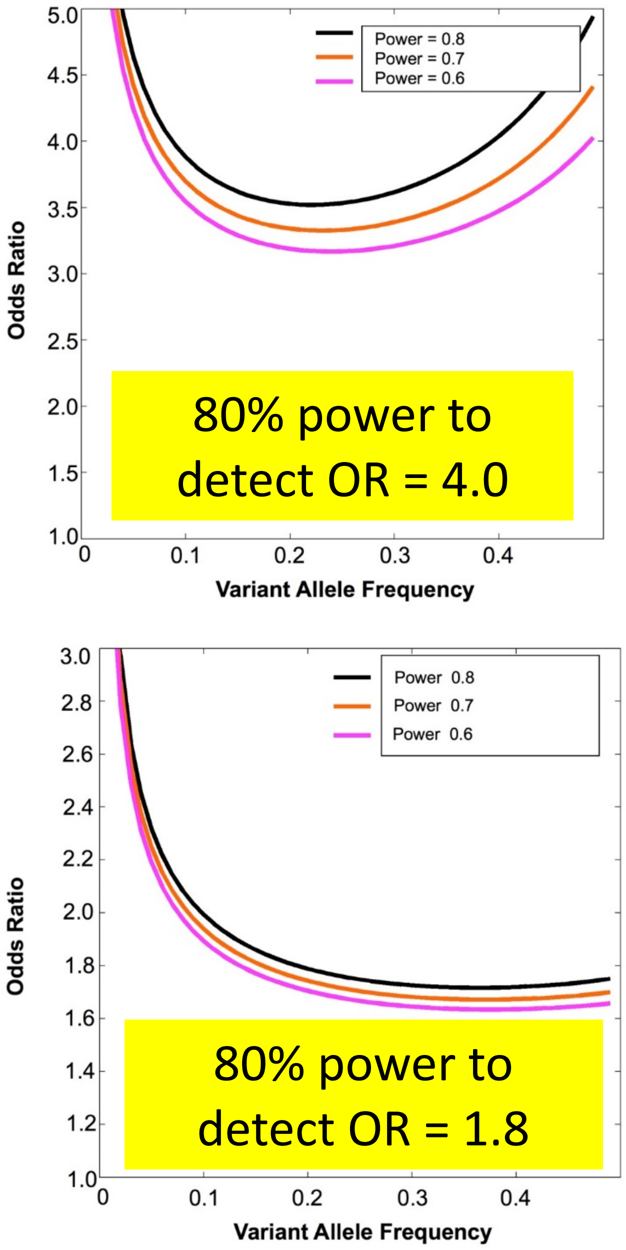

GWAS, genome-wide association study; MPL, moderate prolonged lymphopenia; OR, odds ratio; SPL, severe prolonged lymphopenia.

Supplementary Figure 2. Manhattan plot of *p* values for ~1 million SNPs (minor allele frequency ≥0.05) in a GWAS of SPL ($n = 42$) versus no lymphopenia ($n = 1{,}052$).

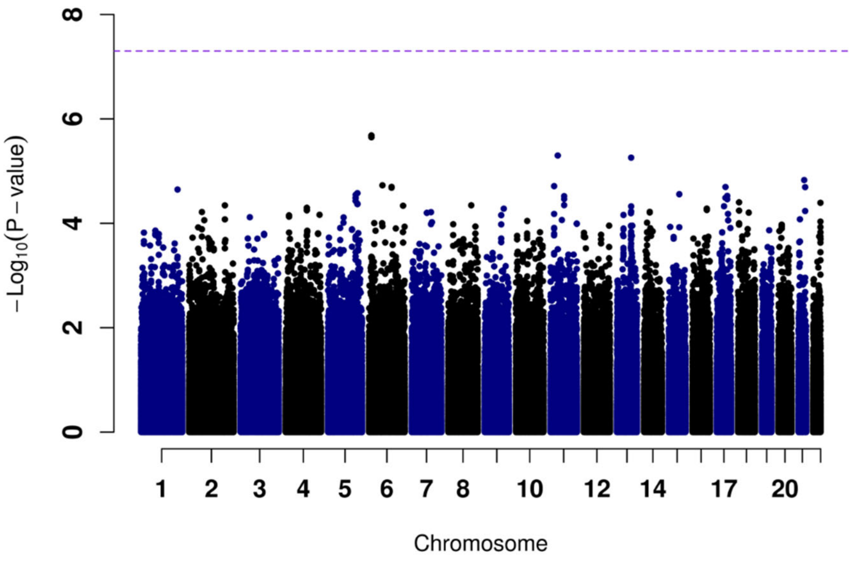

GWAS, genome-wide association study; SNP, single nucleotide polymorphism; SPL, severe prolonged lymphopenia.

Supplementary Figure 3. Quantile-quantile plot of observed versus expected *p* values for ~1 million SNPs (minor allele frequency ≥0.05) in a GWAS of SPL (*n* = 42) versus no lymphopenia (*n* = 1,052).

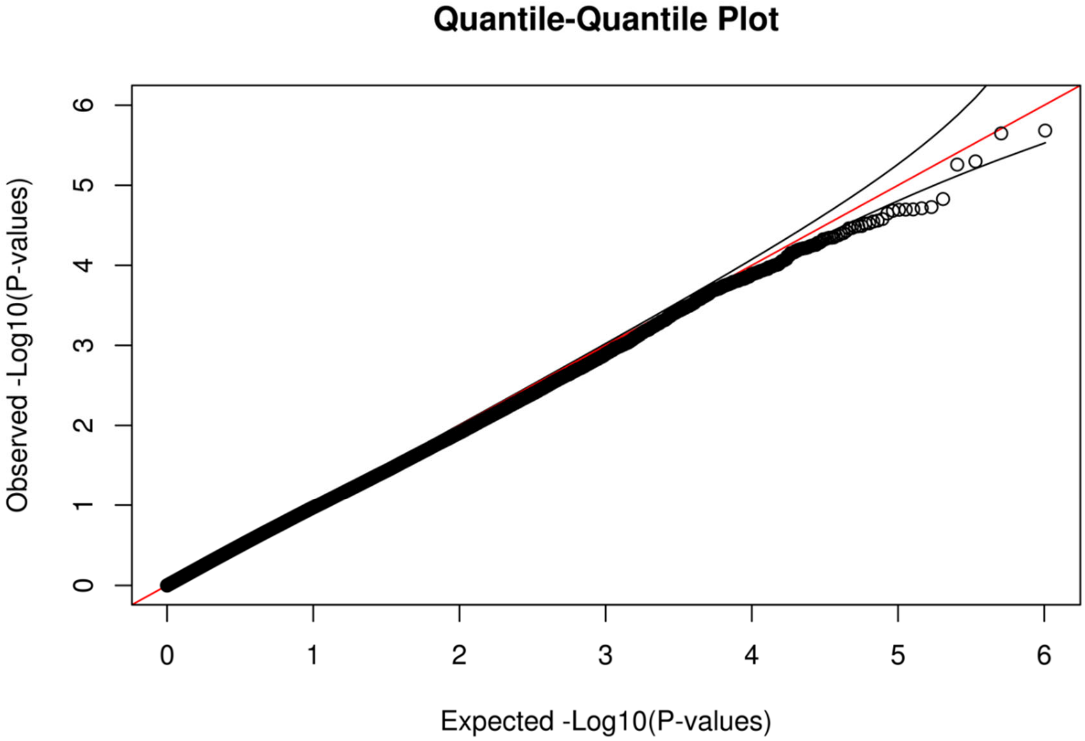

GWAS, genome-wide association study; SNP, single nucleotide polymorphism; SPL, severe prolonged lymphopenia.

Supplementary Figure 4. Quantile-quantile plot of observed versus expected *p* values for ~1 million SNPs (minor allele frequency ≥0.05) in a GWAS of SPL or MPL (*n* = 206) versus no lymphopenia (*n* = 1,052).

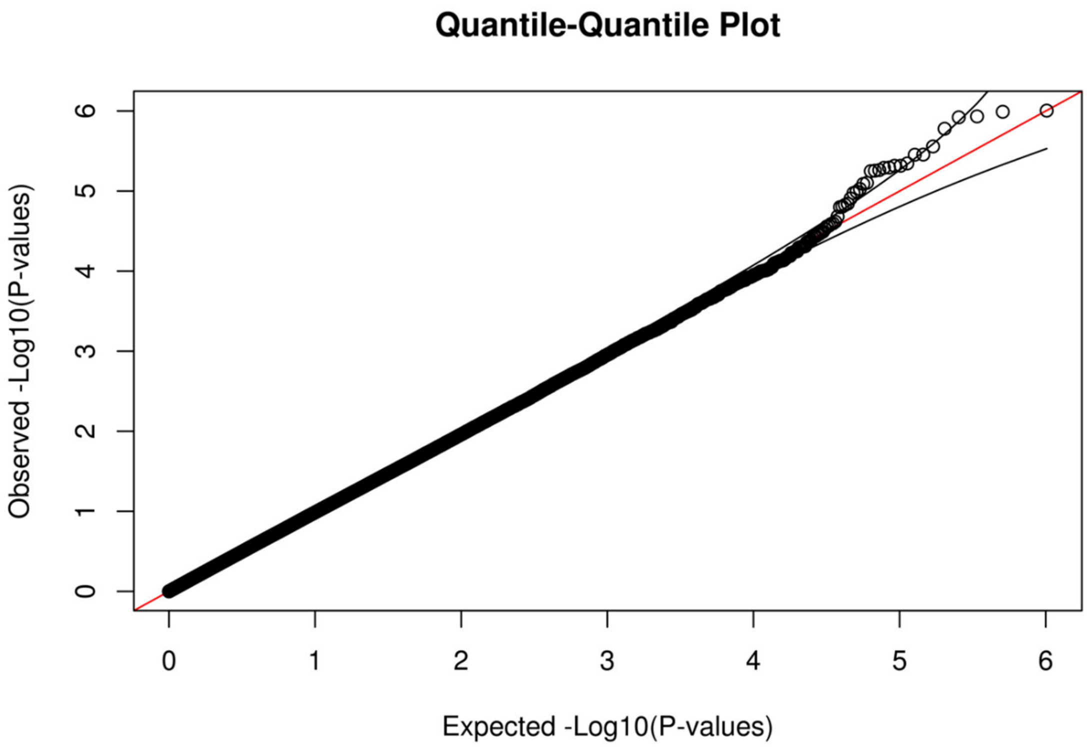

GWAS, genome-wide association study; MPL, moderate prolonged lymphopenia; SNP, single nucleotide polymorphism; SPL, severe prolonged lymphopenia.

Supplementary Figure 5. Nonsignificant peaks in the major histocompatibility complex for **(A)** SPL (*n* = 42) versus no lymphopenia (*n* = 1,052) and **(B)** SPL or MPL (*n* = 206) versus no lymphopenia (*n* = 1,052).

(A)

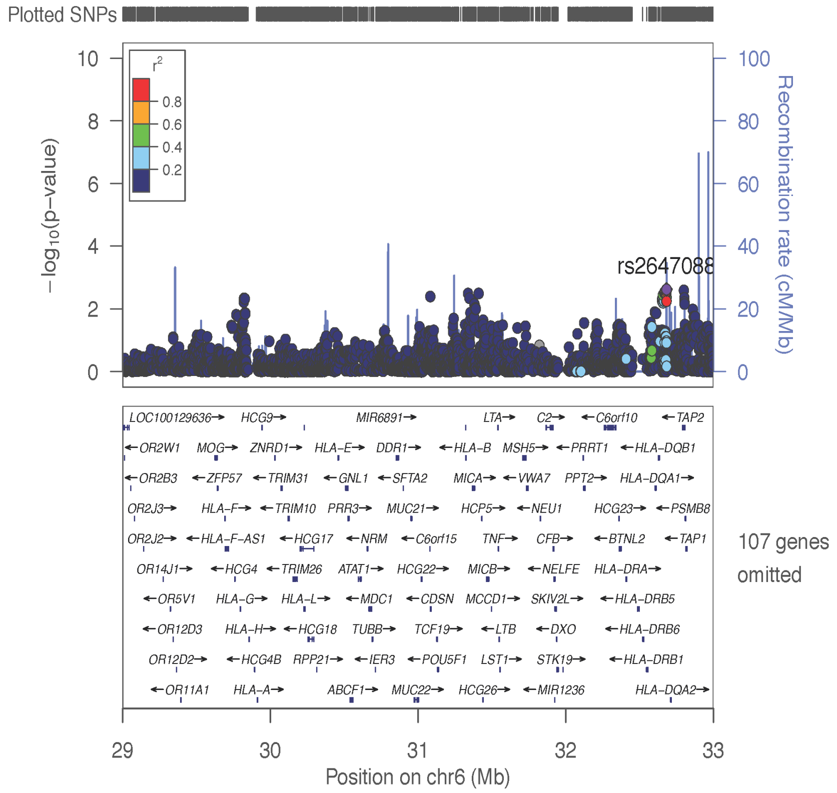

(B)

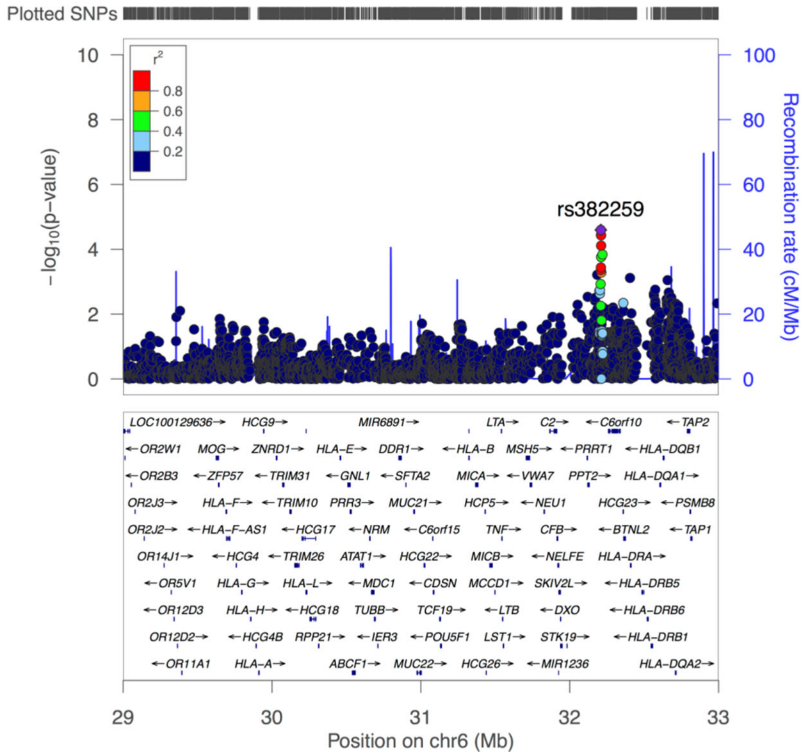

MPL, moderate prolonged lymphopenia; SPL, severe prolonged lymphopenia.

Supplementary Figure 6. Regional association plot of *p* values for SNPs in the major histocompatibility complex gene region on chromosome 6 (29Mb-33Mb) from a GWAS of MS cases with **(A)** SPL versus no lymphopenia (total *n* = 1,094)[a] and **(B)** SPL or MPL versus no lymphopenia (total *n* = 1,258)[b].

(A)

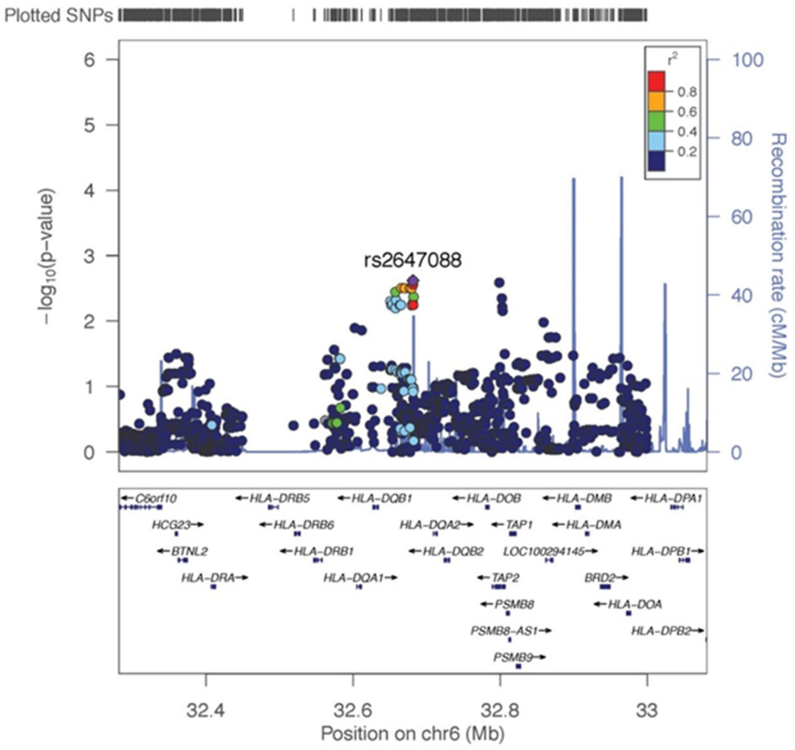

(B)

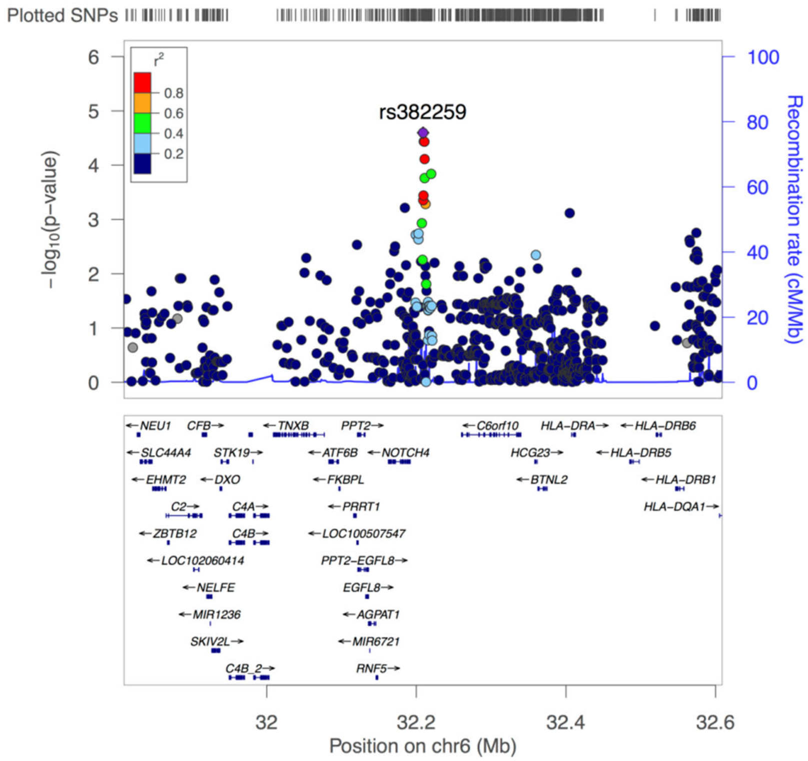

GWAS, genome-wide association study; MPL, moderate prolonged lymphopenia; SNP, single nucleotide polymorphism; SPL, severe prolonged lymphopenia. [a]Odds ratio (OR) 1.7, 95% confidence interval (CI) 1.3–2.2, $p = 2.5 \times 10^{-5}$ at rs382259 (chr6:32,209,027). [b]OR 2.1, 95% CI 1.3–3.5, $p = 2.4 \times 10^{-3}$ at rs2647088 (chr6:32,681,518).

Supplementary Figure 7. Expression of GSTT1 baseline cross-sectional association with lymphopenia severity (0_N: no lymphopenia, 1_M: moderate prolonged lymphopenia, 2_S: severe prolonged lymphopenia).

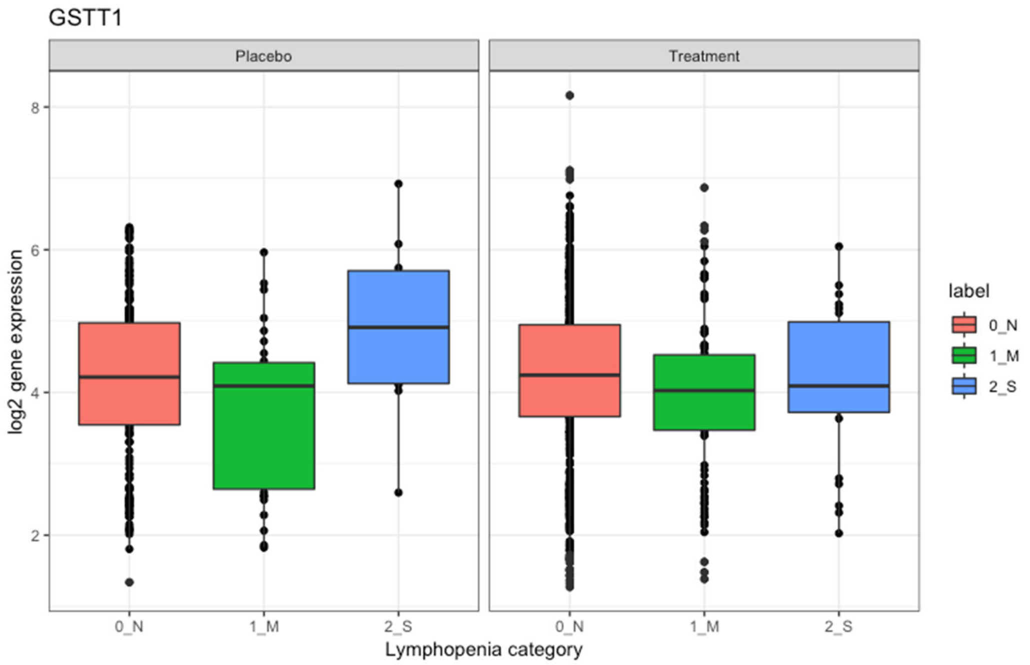

.

GSTT1, glutathione-S-transferase T1. Expression data for the Treatment group was collected at baseline of the DEFINE/CONFIRM trials. Expression data in the placebo group was collected at week 96 of the trials before switching to treatment in the extension study. The association with lymphopenia status was not significant (p = 0.2).

Supplementary Figure 8. Performance metrics comparison of the base (demographic and clinical covariates) and full (including gene expression) models (acc: accuracy; npv: negative predictive value; ppv: positive predictive value; sens: sensitivity; spec: specificity).

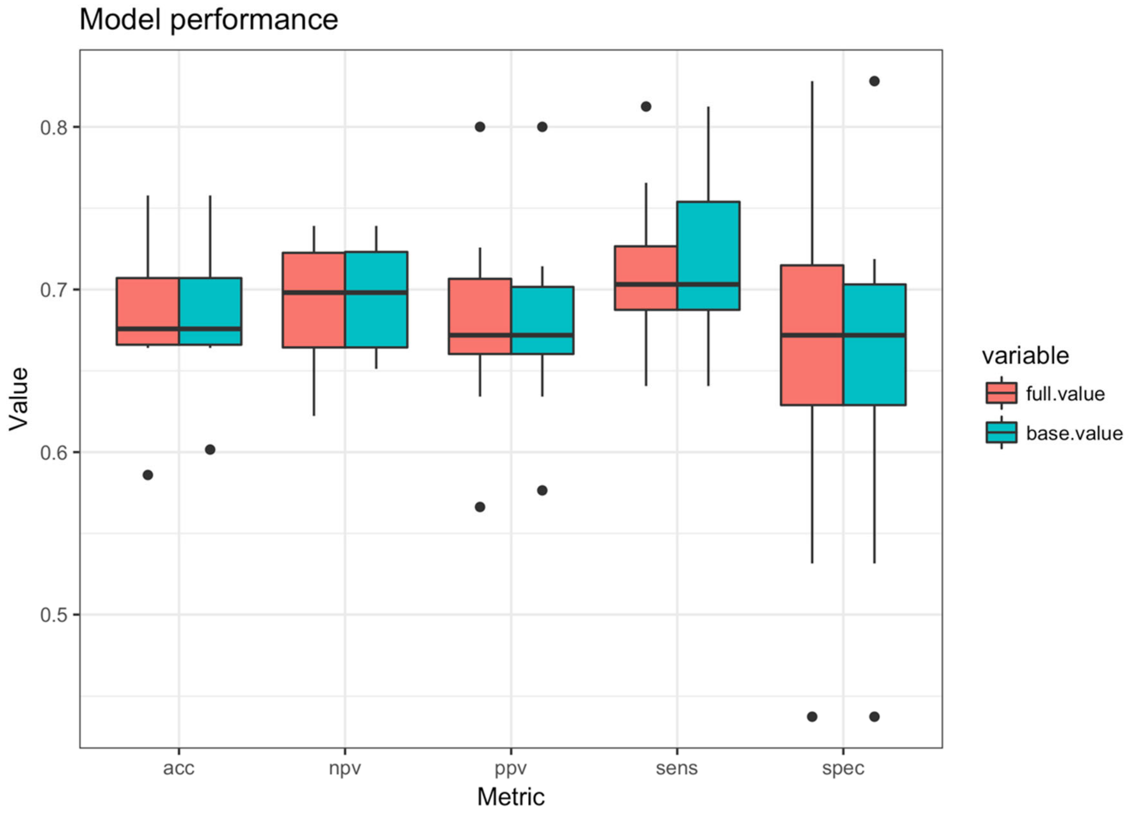

1.2 Supplementary Table

Table S1. Peak association regions (*p* < 1e-5) in the SPL GWAS.

SNP	Allele	*p* Value	OR (95% CI)	Chromosome	Region
rs9392985	A	2.3e-6	3.4 (2.0–5.6)	6	8,266,686–8,266,858
kgp1734375	A	2.1e-6	3.4 (2.1–5.7)		
kgp4224365	G	5.0e-6	2.9 (1.8–4.6)	11	32,368,744–32,375,669
kgp6724547	G	5.5e-6	3.8 (2.1–6.8)	13	91,979,352–91,996,756

GWAS, genome-wide association study; OR, odd ratio; SPL, severe prolonged lymphopenia.

Table S2. Peak association regions in the SPL + MPL GWAS.

SNP	Allele	*p* Value	OR (95% CI)	Chromosome	Region
kgp1734375	A	2.1e-6	3.4 (2.1-5.7)	6	8,266,686-8,266,858
kgp4224365	G	5.0e-6	2.9 (1.8-4.6)	11	32,368,744-32,375,669
kgp6724547	G	5.5e-6	3.8 (2.1-6.8)	13	91,979,352-91,996,756

2 Supplementary Appendix 1

Principal Investigators (DEFINE)
Australia: Michael Barnett, Helmut Butzkueven, Roy Beran, Reynolds Casse, Caron Chapman, Jeannette Lechner-Scott, Richard Macdonell, Mark Paine, Raymond Schwartz; **Austria:** Thomas Berger, Franz Fazekas, Gerhard Ransmayr, Karl Vass; **Belgium:** Shibeshih Belachew, Danny Decoo, Peter Paul De Deyn, Bénédicte Dubois, Luc Vande Gaer, Robert Medaer, Pierrette Seeldrayers, Christian Sindic, Ludo Vanopdenbosch; **Bosnia Herzegovina:** Sanja Grgic; **Canada:** Virender Bhan, Jean-Pierre Bouchart, Suzanne Christie, Amit Bar-Or, François Jacques, Pierre Grammond, Felix Veloso, Galina Vorobeychick; **Croatia:** Vesna Brinar, Vida Demarin, Josip Rudež,Silva, Soldo-Butković, Ranka Baraba Vurdelja; **Czech Republic:** Zdeněk Ambler, David Doležil, Eva Havrdova, Petr Kanovsky, Eva Meluzinova, Jiří Nova'k, Ivan Rektor, Ondrej Skoda, Marta Vachová; **France:** William Camu, Pierre Clavelou, Antoanela Irène Coman, Christian Confavreux, Gilles Edan, Olivier Gout, Christine Lebrun-Frenay, Anne- Caroline Papeix, Patrick Vermersch; **Germany:** Katrin Bachus-Banaschak, Martin Berghoff, Florian Bethke, Hans-Jürgen Boldt, Andrew Chan, Hans-Christoph Diener, Ilonka Eisensehr, Peter Emrich, Bernd Griewing, Judith Haas, Christoph Heesen, Fedor Heidenreich, Frank Hoffmann, Andreas Hufnagel, Ingo Kleiter. Wilfried Lüer, Silke Marckmann-Böenke, Martin Marziniak, Patrick Oschmann, Thorsten Rosenkranz, Hauke Schneider, Veneta Siefjediers, Joachim Springub, Martin Stangel, Florian Stögbauer, Florian Then Bergh, Klaus Tiel-Wilck, Konstanze Tinschert; **Greece:** Nikolaos Grigoriadis, Clementine Karageorgiou, Athanssios Kyritsis, Alexandros Papadimitriou, Thomas Thomaides, Nicholas Vlaikidis; **Guatemala:** David Yaxcal Chon, Luis Fernando Salguero Gonzalez, Hugo Alfredo Ordoñez Sarg; **India:** CS Agrawal, Madhuri Behari, Velmurugendran Cu , Shamsher Dwivedee, Sudhir Kothari, Rahul Kulkarni, Suman Kushwaha, AK Meena, MM Mehndiratta, Subhash Chandra Mukherjee, Lekha Pandit, Arun Shah, Rakesh Shukla, K Vijayan; **Israel:** Anat Achiron, Shlomo Flechter, Ron Milo, Adi Vaknin-Dembinsky; **Italy:** Carlo Pozzilli; **Mexico:** José Luis Oropeza de Alba, Freddy Guillermo Castro Farfan, Ricardo Alberto Rangel Guerra, Ildefonso Rodriguez Leyva, Raúl Arcega Revilla, Jose Flores Rivera; **Moldova:** Mihail Gavriliuc, Stanislav Groppa, Olesea Odainic; **Netherlands:** Raymond Hupperts, E.A.C.M. Sanders, **New Zealand:** Deborah Mason, Paul Timmings, Ernest Willoughby; **Poland:** Anna Czlonkowska, Malgorzata Dorobek, Wieslaw Drozdowski, Hanka Hertmanowska, Waldemar Fryze, Magdalena Kleczkowska, Jan Kochanowicz, Hubert Kwieciński, Przemyslaw Nowacki, Ryszard Podemski, Andrzej Potemkowski, Krzysztof Selmaj, Zbigniew Stelmasiak, Andrzej Wajgt, Tomasz Zielinski; **Republic of Macedonia:** Vera Daskalovska; **Romania:** Alexandru Ovidiu Bajenaru, Fior Dafin Muresanu, Sanda Maria Nica, Mihaela Adriana Simu; **Serbia:** Svetlana Miletić Drakulić, Congor Nadj, Dragana Obradovic, Slobodan Vojinovic; **Slovakia:** Edita Kahancová, Viliam Krajnak, Egon Kurca, L'ubomir Lisý, Peter Turčáni; **South Africa:** Frans Badenhorst, Judy Green, Jeannine Heckmann, Michael Isaacs; **Switzerland:** Ludwig Kappos, Michael Linnebank, Stefanie Müller; **Ukraine:** Nataliya Buchakchyys'ka, Alla Goloborodko, Tetyana Kobys, Volodymyr Lebedynets, Nataliya Lytvynenko, Tetyana Nehrych, Igor Pasyura, Tatyana Ryabichenko, Nataliya Voloshina; **United Kingdom:** Martin Duddy, Clive Hawkins, Richard Nicholas, Jacqueline Palace, Basil Sharrack, Eli Silber, Ben Turner; **United States:** Ann Camac, Warren Chumley, Heidi Crayton, Shanker Dixit, Jeffrey English, Keith Edwards, P. Steven Freedman, Suzanne Gazda, Lawrence Goldstick, Norman Gordon, Christopher Gottschalk, Erica Grazioli, Bart Grelinger, Barbara Green, Ghazala Hayat, Barry Hendin, Todd Janus, Lily Jung Henson, Lawrence Goldstick, William Grainger, Mark Gudesblatt, Ajay Gupta, Afif Hentati, Craig Herrman, Arthur Itkin, Lloyd Kasper, Ellen Lathi, Ellen Mowry, Gareth Parry, Allan Perel, T. Hemanth Rao, Peter Riskind, Syed Rizvi, Michael Rossen, Howard Rossman,

Thomas Scott, S. James Shafer, James Storey, Jr., Ben Thrower, Carlo Tornatore, William Tosches, Lori Trefts, Jr., Anthony Turel, Jr., Bianca Weinstock-Guttman.

Principal Investigators (CONFIRM)

Australia: Christopher Kneebone; **Belarus:** Aliaksandr Fedulau; **Belarus:** Elena Mikhailova; **Belarus:** Sergey Likhachev; **Belarus:** Halina Naumova; **Belgium:** Luc Vande Gaer, Danny Decoo, Christian Sindic; **Bosnia Herzegovina:** Sanja Grgic, Osman Sinanovic, Enra Mehmedika Suljic; **Bulgaria:** Dimitar Georgiev, Lyubomir Haralanov, Sonyia Ivanova, Dimitar Minchev, Ivailo Tournev, Paraskeva Stamenova, Nadezhda Deleva, Zahari Zahariev, Ivan Manchev, Elena Vacheva; **Canada:** Amit Bar-Or, Marcelo Kremenchutzky, Felix Veloso, Norbert Witt, Gregg Blevins; **Costa Rica:** Alexander Parajeles Vindas, Roberto Vargas Howell; **Croatia:** Silva Soldo-Butković, Josip Rudež, Mario Habek, Ranka Baraba Vurdelja; **Czech Republic:** Eva Havrdova, David Doležil, Daniel Vaclavik, Jiří Nova'k; **Estonia:** Katrin Gross-Paju, Katrin Antsov, Sulev Haldre, Alla Palu, Toomas Toomsoo; **France:** William Camu, Jean Pelletier, Pierre Labauge, Marc Debouverie, Gilles Defer, Jérôme De Seze, Thibault Moreau, Abdullatif Al Khedr, Lucien Rumbach; **FYR Macedonia:** Vera Daskalovska; **Germany:** Harald Landefeld, Sabine Masri, Sebastian Schimrigk, Björn Tackenberg, Ilonka Eisensehr, Frank Hoffmann, Bernd Kieseier, Wilfried Lüer, Heike Benes, Christine Paschen, Tobias Derfuß, Michael Sailer, Brigitte Storch-Hagenlocher, Achim Berthele, Patrick Oschmann, Klemens Angnstwurm, Reinhard Hohlfeld, Gerd Reifschneider, Klaus Tiel-Wilck, Gereon Nelles, Hans-Jürgen Boldt, Peter Emrich, Boris-Alexander Kallmann, Wolfgang Feneberg, Angelika Christopher, Reinhard Hüntemann, Mechthild Spiegel-Meixensberger; **Greece:** Thomas Thomaides, Nicholas Vlaikidis, Clementine Karageorgiou, Panagiotis Papathanasopoulos; **India:** Man Mohan Mehndiratta, Krishnan Vijayan, Yash Pal Singh, Suresh Kumar Radhakrishnan, Deepak Arjundas, Rangasetty Srinivasa, Amitabha Ghosh, Rahul Vitthal Kulkarni, Shalin Dipinkumar Shah, Joy Dev Mukherji, Shankara Nellikunja, Madhuri Behari, Gagandeep Singh, Pahari Ghosh, Nasli Rustom Ichaporia, Prahlad Kumar Sethi, Neeta Abhay Mehta, Usha Kant Misra, Maneesh Kumar Singh, Dheeraj Khurana, Abdu Salem; **Ireland:** Michael Hutchinson, Bernard Sweeney; **Israel:** Ronit Gilad, Radi Shahien; **Latvia:** Anita Paegle; **Mexico:** Guillermo Punzo, Jose Santos, Sandra Quiñones, Miguel Angel Macias, Bruno Estañol, Juan Escamilla, Neyla Lopez, Mariela Renteria, Cesar Delgado; **Moldova:** Olesea Odainic, Stanislav Groppa, Mihail Gavriliuc; **New Zealand:** Paul Timmings, **Poland:** Wieslaw Drozdowski, Waldemar Fryze, Jan Kochanowicz, Dr Anna Kaminska, Krzysztof Selmaj, Andrzej Wajgt, Magdalena Kleczkowska, Przemyslaw Nowacki, Anna Czlonkowska, Zbigniew Stelmasiak, Ryszard Podemski, Malgorzata Dorobek, Hanka Hertmanowska, Krystyna Pierzchala, Tomasz Zielinski, Andrzej Szczudlik, Andrzej Tutaj, Jacek Losy, Andrzej Potemkowski, Walenty Nyka, Magdalena Kapelusiak-Pielok; **Romania:** Valentin Ionescu-Dimancea, Rodica Balasa, Petru Mihancea, Cristian Popescu, Liviu Codrut Protosevici; **Serbia:** Slobodan Vojinovic, Svetlana Miletić Drakulić, Ranko Raicevic, Congor Nadj; **Slovakia:** Peter Turčáni, Edita Kahancová, Egon Kurca, L'ubomir Lisý; **Spain:** Xavier Montalbán, Guillermo Izquierdo, Rafael Arroyo, Jose Maria Prieto, Oscar Fernández, Celia Oreja-Guevara, Fernando Sanchez Lopez, Cristina Guijarro; **Ukraine:** Nataliya Voloshina, Igor Pasyura, Borys Palamar, Tetyana Nehrych, Tetyana Kobys, Nataliya Lytvynenko, Alla Goloborodko, Nataliya Buchakchyys'ka, Volodymyr Lebedynets, Tatyana Ryabichenko, Grygory Kushnir, Sergii Moskovko, Galyna Chmyr; **United States:** Mary Forester, Ricardo Ayala, James Voci, Stanley Krolczyk, Braeme Glaun, Robert Smith, Giles Crowell, Revere Philip Kinkel, Malti Patel, Tamara Miller, Gabriel Pardo, Stephen Asher, Christopher LaGanke, Donald Ayres, Matthew Baker, Mitzi Williams, William Sheremata, Alberto Vasquez, Mark Janicki, George Garmany, Jr., Richard Hull, David Steiner, Joseph Herbert, Keith Edwards, Robert Fox, Bhupendra Khatri, Michael Levin, David Mattson, Angela Applebee, Joseph Phillips, Jr., Mary Ann Picone, Warren Felton III, Edward Fox,

Michelle Apperson, Scott Gold, Mariko Kita, Harold Moses, Jr., Robert Shin, John Rinker, II, George Hutton, Lauren Krupp, Patricia Fodor, John Foley, Suzanne Gazda, William Honeycutt, Galen Mitchell, Saud Sadiq, Brian Steingo, Dina Jacobs, Steven Freedman, Bianca Weinstock-Guttman, Sharon Lynch, Anand Vaishnav, Sibyl Wray, Samuel Hunter, Christopher Luzzio, John Huddlestone, Stanley Cohan, Angel Chinea, Daniel Giang, Richard Shubin, Donald Negroski, Allan Perel, Michael Stein, Allan Herskowitz, Jonathan Warach, Daniel Mikol, Roberto Bomprezzi, Geoffery Eubank, Jonathan Licht, Herman Sullivan, T. Hemanth Rao, Stephen Newman, Stuart Silverman, Mark Gudesblatt, William Sunter, Jr., Alireza Minagar, Kottil Rammohan, Malcolm Gottesman, John Schaeffer, Walter Carlini, Lee Stein, Richard Buckler, S. Ausim Azizi, Brendan Bauer, Corey Ford.
